# 

**Published:** 1861-02

**Authors:** 


### CONTENTS.

### ORIGINAL COMMUNICATIONS.

PAGE.
ARTICLE I.—Diphtheritis or Diphtheritic Sorethroat. By T. A. Means, M. D., Memphis, Tenn., ........ 317
ARTICLE II.—Case of Difficult Diagnosis. By D. C. O'Keefe, Atlanta, Ga., ........ 338
ARTICLE III.—Correspondence, ........ 343

### SELECTIONS.

Report of several Cases of Dipththeria. B. S. T. Newman, M. D., of St. Louis, ........ 346
Enteric Fever. By John Richard Wardell, M. D., Editor of the M. R. C. P., ........ 352
Diphtheria. By R. W. I'Anson, M. D., of Dinwiddie Co., Va., ........ 363
Digitalis in the Treatment of Mania a Potu. By Dr. G. C. Catlett, 367

### EDITORIAL AND MISCELLANEOUS.

Who is the "Selfish Demagogue?" ........ 373
Digitalis in Puerperal Fever, ........ 376
Leprosy, Syphilis, Struma, ........ 376
Strychnine in Typhoid Fever, ........ 377
Preservation of Flowers, ........ 377
List of Payments, ........ 377
Register of Meteorological Observations, ........ 378

